# Evaluating the safety, effectiveness and acceptability of treatment of incomplete second-trimester abortion using misoprostol provided by midwives compared with physicians: study protocol for a randomized controlled equivalence trial

**DOI:** 10.1186/s13063-019-3490-5

**Published:** 2019-06-21

**Authors:** Susan Atuhairwe, Josaphat Byamugisha, Marie Klingberg-Allvin, Amanda Cleeve, Claudia Hanson, Nazarius Mbona Tumwesigye, Othman Kakaire, Kristina Gemzell Danielsson

**Affiliations:** 1 Department of Obstetrics and Gynecology, Makerere University, Kampala, Uganda; 2 Mulago National Referral Hospital, Kampala, Uganda; 3 Department of Women and Children’s Health, Karolinska Institutet, Stockholm, Sweden; 4 Karolinska University Hospital, Stockholm, Sweden; 5 School of Education, Health and Social Sciences, Dalarna University, Falun, Sweden; 6 Department of Disease Control, London School of Hygiene and Tropical Medicine, London, UK; 7 Department of Public Health Sciences, Karolinska Institutet, Solna, Sweden; 8 Department of Epidemiology & Biostatistics, School of Public Health, Makerere University, Kampala, Uganda

**Keywords:** Post abortion care, Misoprostol, Second trimester, Task sharing, Incomplete abortion, Contraception, Uganda

## Abstract

**Background:**

A large proportion of abortion-related mortality and morbidity occurs in the second trimester of pregnancy. The Uganda Ministry of Health policy restricts management of second-trimester incomplete abortion to physicians who are few and unequally distributed, with most practicing in urban regions. Unsafe and outdated methods like sharp curettage are frequently used. Medical management of second-trimester post-abortion care by midwives offers an advantage given the difficulty in providing surgical management in low-income settings and current health worker shortages. The study aims to assess the safety, effectiveness and acceptability of treatment of incomplete second-trimester abortion using misoprostol provided by midwives compared with physicians.

**Methods:**

A randomized controlled equivalence trial implemented at eight hospitals and health centers in Central Uganda will include 1192 eligible women with incomplete abortion of uterine size > 12 weeks up to 18 weeks. Each participant will be randomly assigned to undergo a clinical assessment and treatment by either a midwife (intervention arm) or a physician (control arm). Enrolled participants will receive 400 μg misoprostol administered sublingually every 3 h up to five doses within 24 h at the health facility until a complete abortion is confirmed. Women who do not achieve complete abortion within 24 h will undergo surgical uterine evacuation. Pre discharge, participants will receive contraceptive counseling and information on what to expect in terms of side effects and signs of complications, with follow-up 14 days later to assess secondary outcomes. Analyses will be by intention to treat. Background characteristics and outcomes will be presented using descriptive statistics. Differences between groups will be analyzed using risk difference (95% confidence interval) and equivalence established if this lies between the predefined range of − 5% and + 5%. Chi-square tests will be used for comparison of outcome and *t* tests used to compare mean values. *P* ≤ 0.05 will be considered statistically significant.

**Discussion:**

Our study will provide evidence to inform national and international policies, standard care guidelines and training program curricula on treatment of second-trimester incomplete abortion for improved access.

**Trial registration:**

ClinicalTrials.gov, NCT03622073. Registered on 9 August 2018.

**Electronic supplementary material:**

The online version of this article (10.1186/s13063-019-3490-5) contains supplementary material, which is available to authorized users.

## Background

Globally, 303,000 maternal deaths occurred in 2015 [1], with 11% of maternal deaths and a large number of morbidities attributable to consequences of unsafe abortion [2–4]. More than 97% of unsafe abortions occur in sub-Saharan Africa, with the rates of unplanned pregnancy ending in an abortion increasing in low-income settings while decreasing in high-income countries [4]. Reasons behind the high unsafe abortion rates in low-income settings are restrictive abortion laws, high unmet need for contraception and low women’s status [3, 5].

Post-abortion care (PAC), defined as the comprehensive treatment of women presenting after spontaneous or induced, safe or unsafe abortion, is an effective intervention to decrease maternal mortality globally [6]. PAC comprises five key elements: emergency treatment, family planning, counseling, other reproductive and related health services, and community–service provider partnerships [7]. Evidence shows that PAC services can be safely offered by midwives in the first trimester [8, 9]. Evidence is lacking on whether PAC can also be safely offered by midwives in the second trimester particularly in rural settings of low-income countries where physicians are scarce, such as Uganda.

Uganda has a high total fertility rate (5.4 children per woman), a low contraceptive prevalence rate of 39% and an unmet need for contraception of 28% [10]. More than half of all pregancies are unintended due to insufficient knowledge of and misinformation about contraception, inaccessibility to family planing services and ineffective contraceptive use [11]. Multiple obstacles to contraceptive use, such as misconceptions and fears related to contraception, gender–power relations, sociocultural factors and health care service barriers have been cited [12, 13]. Induced abortion in Uganda is restricted and legally permitted only to save a woman’s life [14]. As a result, women often resort to unsafe abortion “that’s either performed by a person lacking the necessary skills or in an environment that does not conform to minimal medical standards” [6]. Of the estimated 314,304 women who undergo unsafe abortions each year, about 128,682 experience complications with only 85,000 obtaining care [15]. Consequently, it is estimated that complications from spontaneous and unsafe abortions contribute to 8% of maternal mortality in Uganda [16]. Post-abortion complications are exacerbated by lack of resources, delayed care-seeking and harmful abortion practices [17]. In some instances, women will fear accessing care due to stigma that is intrinsic, or from family members, community providers and even health providers [18]. Although abortion in Uganda is restricted, treatment of post-abortion complications is a critical component of emergency obstetric care services offered throughout the spectrum of health facilities from Health Centre IIs to hospitals [19, 20]. A study in 2014 cited midwives as the main implementers of PAC in the first trimester, often only consulting a physician when a complication arises [21].

Low-income countries like Uganda suffer from shortages and an unequal distribution of trained health care providers. Almost two thirds of all physicians work in the urban regions, which serve less than one third of the population [20]. While the Ministry of Health (MOH) policy in Uganda allows both physicians and midwives to treat women with first-trimester incomplete abortions, it restricts management of second-trimester incomplete abortion to physicians [19]. The standard treatment for bleeding following an abortion in the first trimester is either misoprostol or manual vacuum aspiration, while sharp curretage by a doctor is done for women in the second trimester. The need for a surgical intervention in a setting where there is a scarcity of trained providers, sterile equipment and poor access to high-level centers limits women’s access to safe PAC, especially in remote areas. Observations in clinical practice show that the number of incomplete abortions in the second trimester are at least as high as in the first trimester in Uganda.

Evidence is compelling that the prostaglandin E1 analogue misoprostol is effective medication for first-trimester incomplete abortions [8, 22], and that there is no difference whether provided by physicians or midwives [8, 23, 24]. The choice of an equivalence trial design is to provide evidence that use of misoprostol for PAC in the second trimester when administered by physicians or midwives is equally safe, effective and acceptable. In view of the severe shortage of medical staff in rural areas and importance of task sharing to improve productivity and efficiency within a health system [21, 25, 26], the overall aim of the study is to compare the safety, effectiveness and acceptability outcomes of treatment for incomplete abortion using misoprostol when provided by midwives versus physicians in Uganda.

## Methods

### Trial design

**Fig. 1:**
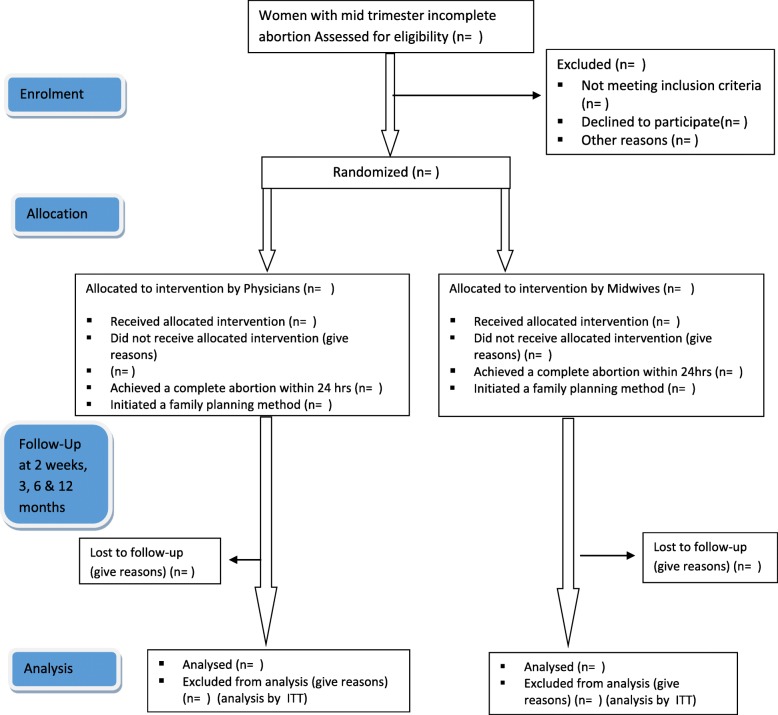
Trial design. ITT intention to treat

The study uses a multicenter randomized controlled equivalence design to compare the management of second-trimester abortion complications with misoprostol to be provided by a midwife or a doctor in a ratio of 1:1 (Fig. 1). The trial follows recommendations of the modified Consort guidelines for equivalence trials [27] and the Helsinki Declaration [28]. A populated Standard Protocol Items: Recommendations for Interventional Trials (SPIRIT) checklist is provided in Additional file 1.

### Study setting

The study is implemented in the central region of Uganda, which has a higher estimated induced abortion rate compared to the national average (52 versus 39 per 1000 live deliveries) and an accompanying huge caseload of women treated for abortion complications [29]. All selected study sites are public health facilities, equipped to provide comprehensive emergency obstetric services in rural, peri-urban and urban areas. They comprise Health Centre IVs (the lowest health care level staffed with both physicians and midwives), district hospitals and a regional referral hospital, all located within 1–2 h from Kampala, the capital city of Uganda.

Health facilities identified for inclusion in the trial have an average caseload of about 90 women with incomplete second-trimester abortions per month and sufficient staff to establish a group of 138 midwives plus other nurse-midwifery cadres at certificate, diploma and degree level and 37 physicians based on mapping of facility records data for January–June 2017, done by one of the researchers (SA). Hospital-based study sites have an average (median) of 5 (6) physicians and 19 (20) midwives, whereas the Health Centre IV study sites have an average (median) of 3 (3) physicians and 13 (16) midwives. We assume that these staff will provide an adequate pool of health providers to conduct the study. The cadre of physicians consist of medical officers and obstetricians/gynecologists with 5 years of medical training and an additional 3 years postgraduate training, respectively. Nursing and midwifery preservice programs in Uganda offer certificate, diploma or degree qualifications after 18 months, 3 years and 4 years of training, respectively. Competences are most often in midwifery and nursing, and there is provision for upgrading to a higher qualification with additional training. In this trial, a midwife is defined as a health provider who has obtained midwifery training competence as part of their preservice or additional training program.

### Eligibility criteria

Women with abortion complications presenting with vaginal bleeding, uterine contractions, uterine size more than 12 weeks but no more than 18 weeks by palpation, history of partial expulsion and an open cervical os will be included in the study. We will exclude women with known age younger than 15 years, allergy to misoprostol, unstable hemodynamic status (systolic blood pressure < 90 mmHg) and shock, signs of pelvic infection and/or sepsis, previous caesarean delivery/uterine scar, suspected extra-uterine pregnancy, perforation of the uterus, injury to the surrounding organs, heavy vaginal bleeding, severe abdominal pain, cervical tear, uterine size ≤12 or > 18 weeks, temperature greater than 38 °C and current molar abortion.

### Recruitment

The study aims to recruit 50 eligible patients monthly over a 24-month period. The health facility staff in all potential recruitment areas, including maternity wards, female wards, emergency wards and outpatient clinics, will be sensitized about the study and encouraged to alert the study team members when a probable case is identified. Notices/posters indicating brief study details and contact persons will be placed at potential recruitment sites of the facility as reminders to staff. All participants are given a modest transport reimbursement of UGX 10,000 (USD 3) for follow-up visits.

### Interventions

#### Enrollment

All women presenting with signs of an incomplete abortion are screened for eligibility by a trained midwife using a predefined checklist. The research assistant obtains written informed consent through a process that involves: giving the participant information concerning the study; providing adequate opportunity for the participant to consider all options; responding to the participant’s questions; ensuring that the participant comprehends this information; obtaining the participant’s voluntary agreement to participate; and continuing to provide information as the participant or situation requires. Women excluded at this point are registered and the reason for exclusion indicated.

#### Procedure

All assessment and care are provided in a way that ensures privacy and confidentiality. Eligible women who consent undergo a clinical assessment by either a midwife (intervention arm) or a physician (control arm). The clinical assessment includes: history taking (sociodemographic information, last menstrual period (LMP), assessment for chronic medical conditions, obstetric and gynecological history, previous use of contraception and details of presenting symptoms); general physical examination and vital signs (pulse, blood pressure and temperature); and abdominal examination to assess the size of the uterus and a vaginal examination to assess cervical opening, bleeding and signs of genital infections.

The World Health Organization (WHO), in their 2012 safe abortion technical and policy guidance, recommend that the misoprostol regimen for second-trimester termination of pregnancy [6] can be used for incomplete abortion management: 400 μg misoprostol sublingual every 3 h up to five doses. Each treatment dose consists of two 200 μg tablets of misoprostol administered sublingually by the allocated health care provider and this is repeated every 3 h for a maximum of five doses or until a complete abortion is confirmed. The allocated health provider to whom a woman is randomized assesses for presence of side effects, vaginal bleeding and uterine contractions, measures blood pressure and pulse and also records any medication given at 3-h intervals before the next dose of misoprostol is given. The participant completes the full course of treatment under direct observation while at the health facility to ensure adherence. When the participant reports an expulsion, a health provider in the allocated arm performs a clinical assessment to diagnose a complete abortion and records the time of completion, and an independent assessor verifies that the main outcome has been achieved. The expelled products of conception are examined to ascertain the type, whether placental tissue only or whole fetus and placental tissue. All women receive oral nonsteroidal anti-inflammatory drugs starting when the first dose of misoprostol is administered and prophylactic oral antibiotics – amoxicillin 500 mg and metronidazole 400 mg every 8 h for 5 days [30].

**Fig. 2:**
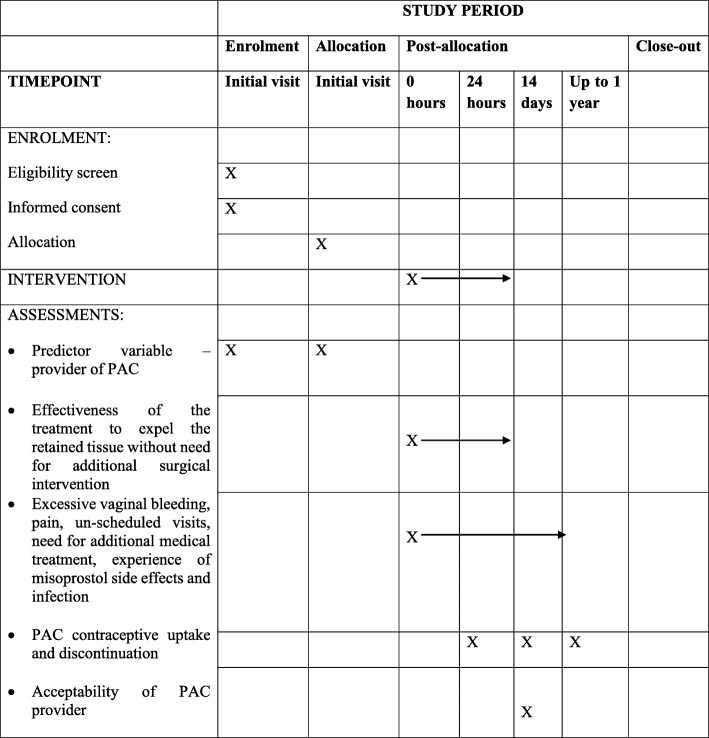
Standard Protocol Items: Recommendations for Interventional Trials figure—participant enrolment and follow-up. PAC post-abortion care

In the case that no complete abortion has been achieved within 24 h from the time the first dose of misoprostol was administered, a doctor performs a surgical method of uterine evacuation using MVA or blunt curettage. Patients can also be discontinued from medical management and surgical management instituted if their condition worsens during treatment or on request. All participants are discharged 4 h after completing the abortion if there are no complications. Before discharge, women receive detailed information regarding abnormal symptoms (fever, foul smelling vaginal discharge, bleeding and pain), when to seek care, and contraceptive counselling and provision, and advised to return for a follow-up visit 14 days after treatment. A detailed description of the study participant timeline is provided in Fig. 2.

### Outcomes

The primary outcome is complete abortion requiring no surgical intervention within 24 h of initiating treatment. A 24-h timeframe was chosen for clinical relevance given that the participants will be monitored at the health facility. Clinical assessment for the primary outcome includes physical and pelvic examination with attainment of a complete abortion based on cessation of uterine cramps and vaginal bleeding and a closed cervical os. An ultrasound scan is not used to diagnose a complete abortion, given its low predicting value to indicate the need of additional surgical intervention. Ultrasound scan measurement of endometrial thickness as an indicator of a complete abortion actually biases toward increased surgical interventions without significantly improving patient outcomes [31].

Secondary outcomes are assessed at the 2-week follow-up visit and include: excessive vaginal bleeding, defined as soaking more than three pads in an hour; abdominal pain, defined as discomfort experienced in the lower abdomen and intensity measured using a visual analogue scale with a minimum score of 0 representing no pain and a maximum score of 10 representing most pain; unscheduled visits, defined as participants presenting at the study site when not expected, need for additional medical treatment and experience of misoprostol side effects; sepsis, defined as presence of fever with temperature greater than 38 °C, pus or offensive vaginal discharge; time from induction (first dose of misoprostol) to completion and total dose of misoprostol given; women’s acceptability of the PAC provider with acceptability defined as positive reporting of treatment experience, recommendation of method to a friend or reuse of same method; and contraceptive uptake and discontinuation over a period of 1 year.

### Data collection methods

The information on history, enrolment, procedures and outcomes is collected using an interviewer-administered pretested structured questionnaire and structured clinical notes. All health workers undertake a standardized 2-day training in misoprostol use for management of incomplete abortion and study procedures. Key areas in the training content include: making a diagnosis of incomplete abortion, use of misoprostol for uterine evacuation, pain relief, post-abortion counseling and values clarification, contraceptive counselling and provision, study procedures, adverse event reporting, danger signs and management of complications. Evaluation of training is done using pre and post knowledge assessments. All research assistants also receive training on research conduct prior to the study. Key areas covered include: research ethical principles, informed consent process, data collection and storage procedures and strategies for retention of study participants. All investigators are trained in good clinical practice. The strategies implemented to promote retention in the study include: clearly informing participants of the study procedure and follow-up schedule, assessing the main outcome before discharge, giving specific follow-up review dates and obtaining at least two phone contacts to ensure communication for follow-up. We conduct continuous process evaluation to ascertain reasons for loss to follow-up, identify and implement context-specific mitigating actions and, in addition, record and analyze reasons for nonadherence or nonretention in the study.

### Sample size

The sample size has been calculated with the objective of establishing the equivalence of misoprostol treatment of incomplete abortion provided by midwives compared with physicians, in terms of the percentage of women not requiring back-up surgical treatment. PASS software version 14 was used for sample size computations for equivalence studies, at a power of 80%, two-sided CI of 95%, set actual difference *D*_1_ range of − 5% to + 5%, smallest difference of 0.001, design effect = 1 and 5% level of significance of results [32]. If a second-trimester abortion treated with misoprostol 400 μg every 6 h administered vaginally has an effectiveness of 90% at 48 h, we assume that the same dose used every 3 h sublingually for second-trimester incomplete abortion would have around 90% effectiveness at 24 h given the faster onset of action for the sublingual route [33]. If both allocation arms have the same success rates within an equivalence margin of 10%, then 566 women will be required in each arm. Considering a loss to follow-up of 5%, 1192 women will be recruited for the study. Table 1 presents the various sample size scenarios, with a δ value of 5% and power of 80% chosen based on the feasibility of accruing the sample size.

**Table 1:** Sample size scenarios

δ (%)	Power = 80%	Power = 90%
4	884 (1768)	1183 (2366)
5	566 (1132)	757 (1514)

### Random allocation

A computer-generated blocked randomization was done by the statistician to generate a randomization list stratified for each study site. The randomization ratio is 1:1 for physicians versus midwives in random blocks of 4–12. The block sizes are not disclosed to the research assistants to ensure concealment. Allocation is concealed using sealed, opaque, sequentially numbered envelopes each containing a serial number and unique study number showing the allocation arm for the individual woman. The randomization list will remain with the statistician throughout the duration of the study and the trial investigators as well as research assistants will not have access to it. Process evaluation is carried out using intermittent check-ups to ensure that the protocol intervention procedures are performed correctly.

### Blinding

Given the nature of the study, the health providers and participants cannot be blinded to the study allocation arm once randomization has occurred. The main outcome assessment is confirmed by an independent assessor who is blinded to the participant’s allocation arm to reduce bias. The data entrants will be blinded to the allocation groups. The Data Safety Monitoring Board (DSMB) will initially be blinded as they conduct the interim analysis, and only unblinded if any safety issues emerge.

### Data management

Data are collected at the study sites in paper case record forms (CRFs) and stored in numerical order in a box file at a secure place. The study coordinator or principal investigator conducts biweekly visits at each of the sites to verify each form for completeness and accuracy. At this point, any missing or inaccurate information is rectified and the checked completed forms brought to the central coordinating center for data entry. Data are double entered into the Epidata version 3.1 study-team-approved soft-copy tool. Provisions (Data Monitoring Plan) have been inserted to check for missing data, with predefined ranges, ensuring use of the correct data format and standardized codes. Any changes (based on discrepancies identified on further cleaning) made to data already entered are only done by authorized personnel and a monthly report indicating the errors cited and corrections made is filed with the project steering committee. Cross-referencing is done with the paper form to ensure completeness of the query correction. A statistician will be involved in analysis of the results.

### Statistical methods

Statistical analysis will be done using Stata version 13. All analyses will be by intention-to-treat (ITT) supplemented by per-protocol analysis. Background characteristics for the two study groups will be described. Categorical data will be presented as proportions and continuous data as means and standard deviations. To determine the differences in background characteristics, chi-square tests will be used for categorical data and Student *t* tests for continuous data. Analysis of the primary outcome between groups will be done using risk difference with 95% confidence intervals and equivalence established if it lies between the predefined range of − 5% and + 5%. *P* ≤ 0.05 will be considered statistically significant. Chi-square tests will be used for comparison of outcomes and Student’s *t* tests used to compare mean values. Comparisons of side effects will be carried out using Fisher’s exact tests.

We will use a generalized linear mixed-effects model with the provider as a fixed effect and the health care facility as a random effect. This will allow us, to some extent, to take into account differences in performance between the sites. Time to completion of abortion will be computed and analyzed using standard survival analysis techniques. Participants will be censored at the time that the main outcome, a complete abortion, is documented or at 24 h if no complete abortion is achieved. Time in this study will be computed in hours. Median times to completion will be derived from Kaplan–Meier estimates of the survival function and the groups will be compared using the log-rank test. Further analysis will be carried out using a Cox proportional hazards model, which establishes factors associated with time to complete abortion in a multivariable model.

### Data monitoring

A group of independent experts with different competences will form a DSMB in order to monitor patient safety and treatment efficacy data while the randomized control trial is ongoing. The board will periodically, at its discretion, review the trial outcome data by the study group plus any other information required and make recommendations to the steering committee. None of the board members is involved in the study implementation or funding of the study. The steering committee is composed of trial investigators as per the title page and two independent members, and chaired by the Head of Department Obstetrics and Gynaecology, Makerere University, Kampala.

Since safety is one of the main issues under study, an interim analysis for safety profile will be done by an independent statistician blinded for treatment allocation when 50% of the data have been collected and entry done. The results will be shared with the DSMB, who will then have a discussion with the steering committee who decide on the continuation of the trial. Annual progress reports are submitted to the Institutional Review Board (IRB) and Uganda National Council for Science and Technology (UNCST) ethical bodies and protocol amendments made if indicated. The trial will not be stopped in case of futility, unless the DSMB advises otherwise and approved by the steering committee. The funders will not have access to the trial data to avoid bias from competing interests.

### Harms

Categories of serious adverse events (SAEs) in this study include death, disability/incapacity, life-threatening sepsis, hospitalization longer than 48 h and blood transfusion for severe hemorrhage. A SAE will be considered unexpected if it is not described in the protocol. SAEs will be recorded as study related only if the participant has signed the consent form and started the intervention. These events will be recorded throughout the time the participant is admitted to the health facility. SAEs occurring after the participant has completed the first follow-up visit will not be reported unless the principal investigator feels that the event may have been caused by the protocol procedure. The PI will promptly inform the institutional review board if a SAE occurs. In the case of physical injury resulting from participation in the study, participants will receive medical treatment at a public health facility until resolution or stabilization.

### Confidentiality

Data tools and instruments are stored securely and access restricted to authorized study team members. The computer used for data storage is password protected. Any results retrieved for interim analysis and monitoring purposes will be password protected and restricted to authorized study team members. Collected data will be backed up on monthly basis and securely stored for a period not exceeding 7 years.

### Dissemination plan

The research team will simplify the study findings and disseminate to the community through local media outlets. Presentations will be made at the Ministry of Health through national Technical Working Group meetings to inform policy-makers. We will publish in international scientific peer-reviewed journals and present at international conferences.

## Discussion

The present research study intends to explore the topics of interest in a coherent and systematic way. The long-term goal of this study is to provide evidence-based information that will contribute to the development of strategies to increase women’s access to safe PAC in low-income contexts where diagnosis and services are constrained. This evidence will provide information to policy-makers in Uganda about women’s reproductive health needs related to availability and improvements in PAC, as well as counseling and contraception for all women. By evaluating the safety and effectiveness of midwives administering misoprostol for treatment of second-trimester incomplete abortion, whose management is currently restricted to physicians, the study is attempting to contribute to the reduction of maternal morbidity and mortality and to mitigate human resource problems in emergency obstetric and gynecological care. A direct economic impact can also be expected due to reduced treatment costs of complications from incomplete abortions.

## Limitations

The study is implemented at study sites with variable client caseloads due to the differing levels of health care provision. We have addressed this using blocked randomization to cater for the uneven number of participants from each site. The health care providers to whom women are randomized work closely at the clinic and within the same environment, which might affect the outcome. Health care providers’ years of working experience might differ between the two groups of midwives and doctors and by individual. Most commonly, midwives working in rural area have longer work experience than doctors. In addition, the health provider’s individual attitude and experiences of using misoprostol might further impact the outcome. All health providers at the participating health facilities will undergo standardized training to strengthen the internal validity.

## Trial status

Protocol version number: PACII 01, issued 17 November 2017. Participant recruitment began on 14 August 2018, and the approximate date recruitment will be completed is 15 August 2020.

## Additional files

Additional file 1: SPIRIT checklist (DOC 122 kb)

Additional file 2: Research proposal IRB approval. Approval letter from Makerere University School of Medicine Research and Ethics Committee. (PDF 934 kb)

Additional file 3: UNCST approval. Approval letter from the Uganda National Council of Science and Technology which provides oversight on research done in Uganda. (PDF 190 kb)

## Data Availability

Data collection training materials are available on request. Once the study is completed, the authors plan to make the data available for wider use.

## Abbreviations

CRF: Case record form
DSMB: Data Safety Monitoring Board
ITT: Intention to treat
LMP: Last menstrual period
MOH: Ministry of Health
PAC: Post-abortion care
SAE: Serious adverse event
UNCST: Uganda National Council for Science and Technology
WHO: World Health Organization
