# Supplementary material for: Evaluating the safety, effectiveness and acceptability of treatment of incomplete second-trimester abortion using misoprostol provided by midwives compared with physicians: study protocol for a randomized controlled equivalence trial
Source: Trials. 2019 Jun 21;20:376. doi: 10.1186/s13063-019-3490-5 (PMC6588936; doi:10.1186/s13063-019-3490-5)
Supplement: Supplementary file 3 — UNCST approval. Approval letter from the Uganda National Council of Science and Technology which provides oversight on research done in Uganda. (PDF 190 kb) [file 13063_2019_3490_MOESM3_ESM.pdf]

Uganda National Council for Science and Techn
(Established by Act of Parliament of the Republic of Uganda)
UNCST

Dear SUSAN BALEKE ATUHAIRWE,

I am pleased to inform you that on **13/06/2018**, the Uganda National Council for Science and Technology (UNCST) approved your study titled, **SAFETY, EFFECTIVENESS AND ACCEPTABILITY OF MISOPROSTOL FOR TREATMENT OF INCOMPLETE MID TRIMESTER ABORTION IN UGANDA** . The Approval is valid for the period of **13/06/2018** to **13/06/2021**.

Your study reference number is **HS153ES**. Please, cite this number in all your future correspondences with UNCST in respect of the above study.

Please, note that as Principal Investigator, you are responsible for:

- Keeping all co-investigators informed about the status of the study.
- Submitting any changes, amendments, and addenda to the study protocol or the consent form, where applicable, to the designated local Research Ethics Committee (REC) or Lead Agency, where applicable, for re-review and approval prior to the activation of the changes.
- Notifying UNCST about the REC or lead agency approved changes, where applicable, within five working days.
- For clinical trials, reporting all serious adverse events promptly to the designated local REC for review with copies to the National Drug Authority.
- Promptly reporting any unanticipated problems involving risks to study subjects/participants to the UNCST.
- Providing any new information which could change the risk/benefit ratio of the study to the UNCST for review.
- Submitting annual progress reports electronically to UNCST. Failure to do so may result in termination of the research project.

Please, note that this approval includes all study related tools submitted as part of the application.

Yours sincerely,

Musa Kwehangana
For: Executive Secretary
UGANDA NATIONAL COUNCIL FOR SCIENCE AND TECHNOLOGY
