# Supplementary material for: Evaluating the safety, effectiveness and acceptability of treatment of incomplete second-trimester abortion using misoprostol provided by midwives compared with physicians: study protocol for a randomized controlled equivalence trial
Source: Trials. 2019 Jun 21;20:376. doi: 10.1186/s13063-019-3490-5 (PMC6588936; doi:10.1186/s13063-019-3490-5)
Supplement: Supplementary file 2 — Research proposal IRB approval. Approval letter from Makerere University School of Medicine Research and Ethics Committee. (PDF 934 kb) [file 13063_2019_3490_MOESM2_ESM.pdf]

MAKERERE UNIVERSITY

P.O. Box 7072 Kampala, Uganda
E-mail: rresearch9@gmail.com

Phone: 256 414 533541
Fax: 256 414 541036/0414 532204

COLLEGE OF HEALTH SCIENCES
SCHOOL OF MEDICINE

RESEARCH ETHICS COMMITTEE

November 17, 2017

Dr. Susan Atuhairwe
Department of Obstetrics and Gynecology

Category of review

[X] Initial review
[ ] Continuing review
[ ] Amendment
[ ] Termination of study
[ ] SAEs

Dear Dr. Atuhairwe,

Re: Approval of proposal #REC REF 2017-016

"Safety, effectiveness, and acceptability of Misoprostol for treatment of incomplete mid trimester abortion in Uganda"

Thank you for submitting an application for approval of the above – referenced **proposal**.
The committee reviewed it and granted approval for one year, effective November 17th, 2017. Approval will expire on November 16th, 2018.

Continuing Review
In order to continue work on this study (including data analysis) beyond the expiration date, the School of Medicine Research and Ethics Committee must reapprove the protocol after conducting a substantive, meaningful, continuing review. This means that you must submit a continuing report form as a request for continuing review. To best avoid a lapse, you should submit the request six (6) to eight (8) weeks before the lapse date. Please use the forms supplied by our office.

Amendments
During the approval period, if you propose any change to the protocol such as its funding source, recruiting materials, or consent documents, you must seek School of Medicine Research and Ethics Committee approval before implementing it.
Please summarize the proposed change and the rationale for it in a letter to the School of Medicine Research and Ethics Committee. In addition, submit three (3) copies of an updated version of your original protocol application- one showing all proposed changes in bold or 'track changes,' and the other without bold or track changes.

Reporting
Other events which must be reported promptly in writing to the School of Medicine Research and Ethics Committee include:
Suspension or termination of the protocol by you or the grantor
Unexpected problems involving risk to participants or others

Adverse events, including unanticipated or anticipated but severe physical harm to participants.

Do not hesitate to contact us if you have any questions. Thank you for your cooperation and commitment to the protection of human subjects in research.

Final approval is to be granted by Uganda National Council for Science and Technology.

MAKERERE UNIVERSITY
SCHOOL OF MEDICINE
APPROVED
VALID UNTIL
16 NOV 2018
RESEARCH & ETHICS COMMITTEE
P.O.BOX 7072, KAMPALA

Yours sincerely,

Assoc. Prof. Ponsiano Ocama
Chairperson School of Medicine Research and Ethics Committee
